# Integrative Bioinformatics Analysis Reveals Pathogenesis Biomarkers for Clozapine-Induced Metabolic Syndrome

**DOI:** 10.31083/AP49352

**Published:** 2025-12-22

**Authors:** Yingyi Wang, Haisu Wu, Ruijie Geng, Chongze Wang, Qinyu Lv, Zezhi Li, Zhenghui Yi

**Affiliations:** ^1^Department of Nutritional and Metabolic Psychiatry, The Affiliated Brain Hospital, Guangzhou Medical University, 510370 Guangzhou, Guangdong, China; ^2^Guangdong Engineering Technology Research Center for Translational Medicine of Mental Disorders, 510370 Guangzhou, Guangdong, China; ^3^Division of Psychotic Disorders, Shanghai Mental Health Center, Shanghai Jiao Tong University School of Medicine, 200030 Shanghai, China; ^4^Department of Psychological Medicine, Zhongshan Hospital, Fudan University, 200032 Shanghai, China; ^5^Department of Psychiatry, Huashan Hospital, Fudan University, 200040 Shanghai, China; ^6^Institute of Mental Health, Fudan University, 200032 Shanghai, China

**Keywords:** schizophrenia, antipsychotic agents, clozapine, metabolic syndrome, RNA sequencing

## Abstract

**Objective:**

To explore the molecular mechanisms underlying clozapine-induced metabolic syndrome (MetS) in schizophrenia patients, providing scientific evidence for clinicians to prevent and manage metabolic syndrome during the treatment of psychiatric disorders.

**Methods:**

Ten schizophrenia patients with MetS and ten matched controls were recruited from Shanghai Mental Health Center according to the fourth edition of the Diagnostic and Statistical Manual of Mental Disorders (DSM-IV) criteria for schizophrenia and the 2016 Chinese Adult Dyslipidemia Prevention and Treatment Guidelines for MetS. Peripheral blood RNA sequencing was performed to identify differentially expressed genes (DEGs). Weighted gene co-expression network analysis (WGCNA) and protein-protein interaction (PPI) network were used to pinpoint hub genes. Mendelian randomization (MR) was conducted to validate causal relationship between serum brain-derived neurotrophic factor (BDNF) levels and MetS components.

**Results:**

A total of 1019 DEGs were identified, grouped into eight mRNA modules through WGCNA. Key hub genes included *RP11-611O2.6*, acid phosphatase-like 2 (*ACPL2*), T cell receptor alpha variable 12-2 (*TRAV12-2*), matrix metallopeptidase 8 (*MMP8*), piggyBac transposable element derived 4 pseudogene 1 (*PGBD4P1*), transmembrane protein 261 (*TMEM261*), and *BDNF*, with *BDNF* and *MMP8* further validated by PPI network analysis. MR analysis confirmed a causal association between BDNF levels and MetS risk, reinforcing its role in metabolic dysregulation. Gene Ontology (GO) annotation and pathway enrichment analysis highlighted immune response, morphological changes, and metabolic processes as key biological processes, with pathways such as biological oxidation and defensins significantly enriched.

**Conclusion:**

Significant differences in gene expression are observed between schizophrenia patients with and without MetS. Individual variability in clozapine-induced MetS may be linked to DEGs.

## Main Points

1. Significant differences in gene expression are observed between schizophrenia patients with and without metabolic syndrome (MetS). Individual variability in clozapine-induced MetS may be linked to differentially expressed genes (DEGs).

2. Brain-derived neurotrophic factor (*BDNF*), matrix metallopeptidase 8 (*MMP8*), T cell receptor alpha variable 12-2 (*TRAV12-2*), acid phosphatase-like 2 *(ACPL2*), transmembrane protein 261 (*TMEM261*), piggyBac transposable element derived 4 pseudogene 1 (*PGBD4P1*) and *RP11-611O2.6* were identified as key hub genes in the pathogenesis of clozapine-induced MetS in schizophrenia patients.

3. Mendelian randomization (MR) analysis found that genetically predicted higher serum BDNF levels were causally associated with reduced risks of type 2 diabetes, hyperlipidemia and hypertension.

4. Gene Ontology (GO) annotation and pathway enrichment analysis revealed that clozapine-induced metabolic syndrome primarily involves key biological processes such as immune response, morphological changes, and metabolic processes, with significant enrichment in pathways like biological oxidation and defensins.

## 1. Introduction

Schizophrenia is a severe psychiatric disorder that affects approximately 0.7% of the global population [1]. Second-generation antipsychotic drugs (SGAs) are the primary medications for treating schizophrenia and preventing relapse [2]. Compared to first-generation antipsychotics, SGAs offer significant advantages in effectively controlling both positive and negative symptoms, with fewer extrapyramidal side effects [3]. However, these drugs are not without serious adverse effects. Numerous studies have shown that the use of SGAs is closely associated with the development of metabolic syndrome (MetS) [4, 5, 6, 7]. MetS is a pathological condition characterized by a cluster of metabolic abnormalities, including cardiovascular changes, hypertension, dyslipidemia, weight gain, insulin resistance, and type 2 diabetes, all of which increase the risk of cardiovascular diseases in patients [8]. Among those receiving antipsychotic treatment, the highest rates of MetS are observed in patients treated with clozapine [9, 10]. These metabolic side effects not only impair medication adherence but also substantially increase the risk of schizophrenia relapse, ultimately diminishing both life expectancy and quality of life [11]. Consequently, the metabolic and cardiovascular risks induced by clozapine have become one of the major factors limiting its clinical use. A better understanding of the mechanisms underlying clozapine-induced MetS is crucial for predicting and managing these adverse metabolic effects, and for facilitating more personalized and precise drug selection aimed at reducing metabolic risks and optimizing therapeutic outcomes.

Currently, the mechanism of clozapine-induced MetS remains incompletely understood. At the peripheral level, clozapine affects target organs involved in insulin action and energy regulation pathways, leading to insulin resistance and disturbances in glucose and lipid metabolism [12, 13]. At the central nervous system level, clozapine regulates neurotransmitters, such as dopamine, histamine, serotonin, and neuropeptides, influencing feeding behavior and the satiety center, which promotes the onset and development of MetS in patients [13, 14]. Candidate gene association studies and genome-wide association study (GWAS) have identified several potential susceptibility genes associated with clozapine-induced MetS, including 5-hydroxytryptamine receptor 2A (*HTR2A*), 5-hydroxytryptamine receptor 2C (*HTR2C*), dopamine receptor D2 (*DRD2*), dopamine receptor D3 (*DRD3*), tumor necrosis factor (*TNF*), etc. [15]. Although GWAS has made significant progress in identifying disease-associated genetic variants, it primarily focuses on genetic variations without providing insights into gene expression changes under specific conditions [16]. The development of schizophrenia and MetS is a result of complex interactions between genetic and environmental factors, it is likely that the regulation of downstream gene expression may play a critical role [8, 17]. Transcriptomics, as a powerful tool, can complement GWAS by reflecting the active expression of genes under specific environmental conditions or time frames [18]. By analyzing differentially expressed genes (DEGs), we can identify key regulatory pathways and potential biomarkers associated with clozapine-induced MetS, offering insights into its pathogenesis and potential therapeutic targets.

Therefore, this study aims to recruit schizophrenia patients with long-term clozapine use, using RNA sequencing technology to obtain the complete transcriptome data. We compared gene expression differences between schizophrenia patients with and without MetS to explore the mechanisms underlying clozapine-induced MetS. Our hypothesis is that there are gene expression differences between schizophrenia patients with and without MetS, and that individual variations in clozapine-induced MetS are associated with DEGs.

## 2. Material and Methods

### 2.1 Inclusion and Exclusion Criteria

Chronic schizophrenia patients who had been hospitalized for a long term at the Shanghai Mental Health Center were selected for this study. Inclusion criteria were: (1) diagnosis of schizophrenia according to the fourth edition of the Diagnostic and Statistical Manual of Mental Disorders (DSM-IV); (2) diagnosis of MetS according to the 2016 China Adult Dyslipidemia Prevention and Treatment Guidelines, with three or more of the following conditions: (a) Central or abdominal obesity: waist circumference ≥90 cm for males or ≥85 cm for females; (b) Hyperglycemia: fasting blood glucose ≥6.10 mmol/L, 2-hour blood glucose ≥7.8 mmol/L, and/or a confirmed diagnosis of diabetes under treatment; (c) Hypertension: blood pressure ≥130/85 mmHg and/or a confirmed diagnosis of hypertension under treatment; (d) Fasting triglycerides (TG) ≥1.7 mmol/L; (e) Fasting high-density lipoprotein (HDL) cholesterol <1.0 mmol/L; (3) use of clozapine for ≥2 years; (4) Han Chinese ethnicity; (5) informed consent. Exclusion criteria were: (1) presence of organic brain diseases or dependence on psychoactive substances or alcohol; (2) severe physical illnesses, including serious neurological or cardiovascular diseases, malignant tumors, immunodeficiency, agranulocytosis, acute or chronic renal failure, liver cirrhosis, or active liver disease; (3) endocrine or metabolic disorders, such as hyperthyroidism, hypothyroidism, or Cushing’s syndrome; (4) history of metabolic abnormalities (e.g., diabetes, hypertension, hyperlipidemia) prior to clozapine treatment.

A matched normal control group was selected from the Shanghai Mental Health Center, consisting of individuals diagnosed with schizophrenia according to the DSM-IV criteria but not meeting the MetS diagnostic criteria. Informed consent was obtained from all participants or their legal guardians.

### 2.2 Clinical Assessment and Sample Collection

General clinical data from the participants were collected by the psychiatrist, and their psychiatric status was assessed using the Positive and Negative Syndrome Scale (PANSS) and the Clinical Global Impressions-Severity Scale (CGI-S). Fasting venous blood samples (2 mL) were collected from all participants at 7:00 AM on the same day for RNA sequencing.

### 2.3 Differentially Expressed Genes (DEGs) Screening 

Fragments per kilobase million (FPKM) was used as a measure of transcript or gene expression levels, with the following calculation formula: FPKM = cDNA fragments/(Mapped fragments (millions) × Transcript length (kb)). In this formula, cDNA fragments refer to the number of fragments mapped to a specific transcript, i.e., the number of paired-end reads; Mapped fragments (millions) refer to the total number of fragments mapped to the transcript, expressed in millions (10^6^); and Transcript length (kb) refers to the length of the transcript, expressed in kilobases (10^3^ base pairs). Differential expression analysis between sample groups was performed using the DESeq R package. The false discovery rate (FDR) was calculated by adjusting the significance *p*-values (*p*-value). In the differential expression gene detection process, an FDR threshold of <0.05 was used as the selection criterion. Since differential expression analysis in transcriptome sequencing involves independent statistical hypothesis testing for a large number of gene expression values, which may lead to false positives, the widely accepted Benjamini-Hochberg correction method was applied to adjust the original *p*-values for significance.

### 2.4 Weighted Gene Co-Expression Network Analysis

Weighted gene co-expression network analysis (WGCNA) was conducted using the WGCNA package in R. First, a similarity matrix for each gene pair was calculated, where the co-expression similarity (S_i⁢j_) was defined as the absolute value of the correlation coefficient between nodes* i *and *j*. To establish the soft-thresholding power β, we adhered to the scale-free topology criterion, ensuring that the logarithm of the connection probability log(p(*i*)) is negatively correlated with the logarithm of the number of connections log(*i*), with a correlation coefficient of at least 0.8. The adjacency matrix was then constructed by raising each pairwise correlation to the power of β, effectively weighting the network connections.

Next, to reduce noise and spurious correlations, the adjacency matrix was transformed into a topological overlap matrix (TOM), and the corresponding dissimilarity measure was calculated. A hierarchical clustering dendrogram was generated to represent the hierarchical clustering structure, with each short vertical line representing a gene and each branch corresponding to a module. To identify modules, a dynamic tree-cutting algorithm was applied to the dendrogram. Parameters were set with a minimum module size of 100 and a module merging threshold of 0.15.

Gene assignment to modules was based on module membership (kME), defined as the correlation between the gene’s expression profile and the module eigengene (ME). The ME represents the first principal component of the standardized module expression profile. Genes with kME >0.8 were assigned to colored modules, whereas those not meeting this criterion were grouped into the gray module.

Pearson correlation analysis was conducted to assess the relationships between the identified modules and five metabolic traits. Genes exhibiting the highest gene significance (GS), module membership (MM), and intramodular connectivity were identified as hub genes. These hub genes are pivotal, defining the core characteristics of their respective modules and exhibiting strong correlations with relevant clinical traits.

### 2.5 Protein-Protein Interaction (PPI) Network Analysis and Functional Annotation

Differentially expressed genes were used to construct protein-protein interaction networks using the STRING tool (Version 12.0, https://string-db.org/) with a confidence score threshold of >0.7 for significance. Network analysis was performed using Cytoscape (version 3.7.0; The Cytoscape Consortium, San Diego, CA, USA), and network topological properties were calculated with the NetworkAnalyzer (undirected) tool. Betweenness centrality was computed using the CytoNCA plugin. Hub proteins were identified by integrating the results with WGCNA analysis. To further investigate the role of differentially expressed genes in clozapine-induced MetS and the pathways they participate in, Gene Ontology (GO) functional enrichment, Kyoto Encyclopedia of Genes and Genomes (KEGG) pathway enrichment, and Reactome pathway enrichment analyses were conducted using R and the clusterProfiler package, along with Metascape.

### 2.6 Mendelian Randomization

To infer the causal relationships of genetically predicted serum brain-derived neurotrophic factor (BDNF) levels on MetS components, we performed a two-sample Mendelian randomization (MR) analysis based on three core assumptions: (1) genetic instruments (single-nucleotide polymorphisms, SNPs) must strongly associate with serum BDNF levels; (2) SNPs are independent of confounding factors; and (3) SNPs influence outcomes exclusively through serum BDNF levels, excluding horizontal pleiotropy (**Supplementary Fig. 1**). The GWAS summary statistics of serum BDNF levels were obtained from the GWAS public databases [19], which included 5368 European individuals. Instrumental variables (IVs) for BDNF were selected from GWAS summary statistics under following criteria: (1) SNPs associated with serum BDNF levels at genome-wide significance (*p* < 1 × 10^-5^); (2) linkage disequilibrium (LD) clumping (r^2^ <0.001, clumping window = 1000 kb) to ensure independence; and (3) exclusion of pleiotropic SNPs associated with confounders via PhenoScanner. At last 37 SNPs were selected as instrumental variables for serum brain-derived neurotrophic factor levels (**Supplementary Table 1**). The statistics for the components of MetS were obtained from GWAS public databases, and data on the waist circumference, hypertension, triglycerides, HDL cholesterol and type 2 diabetes were collected. The specific information for data sources is provided in **Supplementary Table 2**.

Analysis included inverse-variance weighted (IVW) regression as the primary method, supplemented by MR-Egger, weighted median, weighted mode and simple mode. Horizontal pleiotropy was evaluated via MR-Egger intercept tests, and heterogeneity was assessed using Cochran’s Q statistic. The leave-one-out analysis was performed to demonstrate the resilience of the findings through sensitivity analysis and examining each SNP [20], which enabled the identification and exclusion of variables that could potentially influence the results. All analyses were performed in R with the TwoSampleMR packages, ensuring harmonization of ancestry and effect alleles between exposure and outcome datasets.

### 2.7 Statistical Analysis

All statistical analyses were performed using SPSS 24.0 software (IBM Corp., Armonk, NY, USA). Categorical variables were presented as frequency (percentage) and analyzed with Fisher’s exact test for intergroup comparisons. The distribution of continuous variables was assessed using the Shapiro-Wilk test: normally distributed variables with homogeneous variances were expressed as mean ± standard deviation and compared using *t*-test, while normally distributed variables with heterogeneous variances were expressed as mean ± standard deviation and analyzed via Welch’s *t*-test with adjusted degrees of freedom; non-normally distributed variables were described as median (Q1, Q3) and analyzed through the Mann-Whitney U test. All statistical inferences adopted a two-tailed α level of 0.05 as the significance threshold.

## 3. Results

### 3.1 Demographic and Clinical Characteristics of the Case and Control Groups

No significant differences were found between the two groups in demographics, clinical features, or clozapine dosage and plasma concentration (*p* > 0.05). Significant differences were observed in waist circumference, blood pressure, fasting glucose, lipids, education duration and PANSS total score (*p* < 0.05), with no differences in CGI-S total score (*p* > 0.05). Detailed demographic and clinical data are shown in Table 1.

**Table 1.** **Demographic and clinical characteristics of the case and control groups**.

Parameter	Case (n = 10)	Control (n = 10)	*t*/Z	*p*
Age (years)	65.80 ± 4.52	66.00 ± 4.11	–0.104	0.919^a^
Gender (male)	6 (60%)	6 (60%)	-	1.000^d^
Duration of illness (years)	40.20 ± 5.88	42.80 ± 6.07	–0.973	0.344^a^
Medication duration (years)	37.00 (35.50, 40.25)	42.00 (38.75, 43.00)	–1.600	0.123^c^
Marital status (married)	4 (40%)	2 (20%)	-	0.628^d^
Education duration (years)	9.00 (9.00, 12.00)	9.00 (9.00, 9.00)	–2.169	0.030^c^
Smoking	3 (30%)	3 (30%)	-	1.000^d^
Alcohol use	0 (0)	0 (0)	-	-
Psychiatric family history	3 (30%)	2 (20%)	-	1.000^d^
Clozapine dosage (mg/day)	152.50 ± 86.96	157.50 ± 81.69	–0.133	0.896^a^
Clozapine level (ng/mL)	333.40 ± 158.95	266.70 ± 134.81	1.012	0.325^a^
Waist circumference (cm)	99.00 ± 6.77	80.60 ± 6.52	6.193	<0.001^a^
SBP (mmHg)	138.40 ± 8.09	115.40 ± 16.97	3.870	0.001^a^
DBP (mmHg)	85.70 ± 7.79	74.20 ± 9.93	2.881	0.010^a^
FPG (mmol/L)	6.78 ± 1.75	4.70 ± 0.45	3.642	0.004^b^
TG (mmol/L)	2.10 (1.97, 3.09)	0.81 (0.71, 1.05)	–3.780	<0.001^c^
HDL cholesterol (mmol/L)	0.88 ± 0.06	1.25 ± 0.21	–5.275	<0.001^b^
PANSS total score	52.00 (50.75, 66.00)	65.50 (60.00, 68.25)	–2.009	0.045^c^
CGI-S total score	5.00 (4.00, 6.00)	6.00 (4.75, 6.25)	–1.245	0.247^c^

SBP, systolic blood pressure; DBP, diastolic blood pressure; FPG, fasting plasma glucose; TG, triglycerides; HDL cholesterol, high-density lipoprotein cholesterol; PANSS, Positive and Negative Syndrome Scale; CGI-S, Clinical Global Impressions-Severity Scale; ^a^*t*-test; ^b^ Welch’s *t*-test; ^c^ Mann-Whitney U test; ^d^ Fisher’s exact test.

### 3.2 Data Preprocessing and Identification of DEGs

**Fig. 1.**
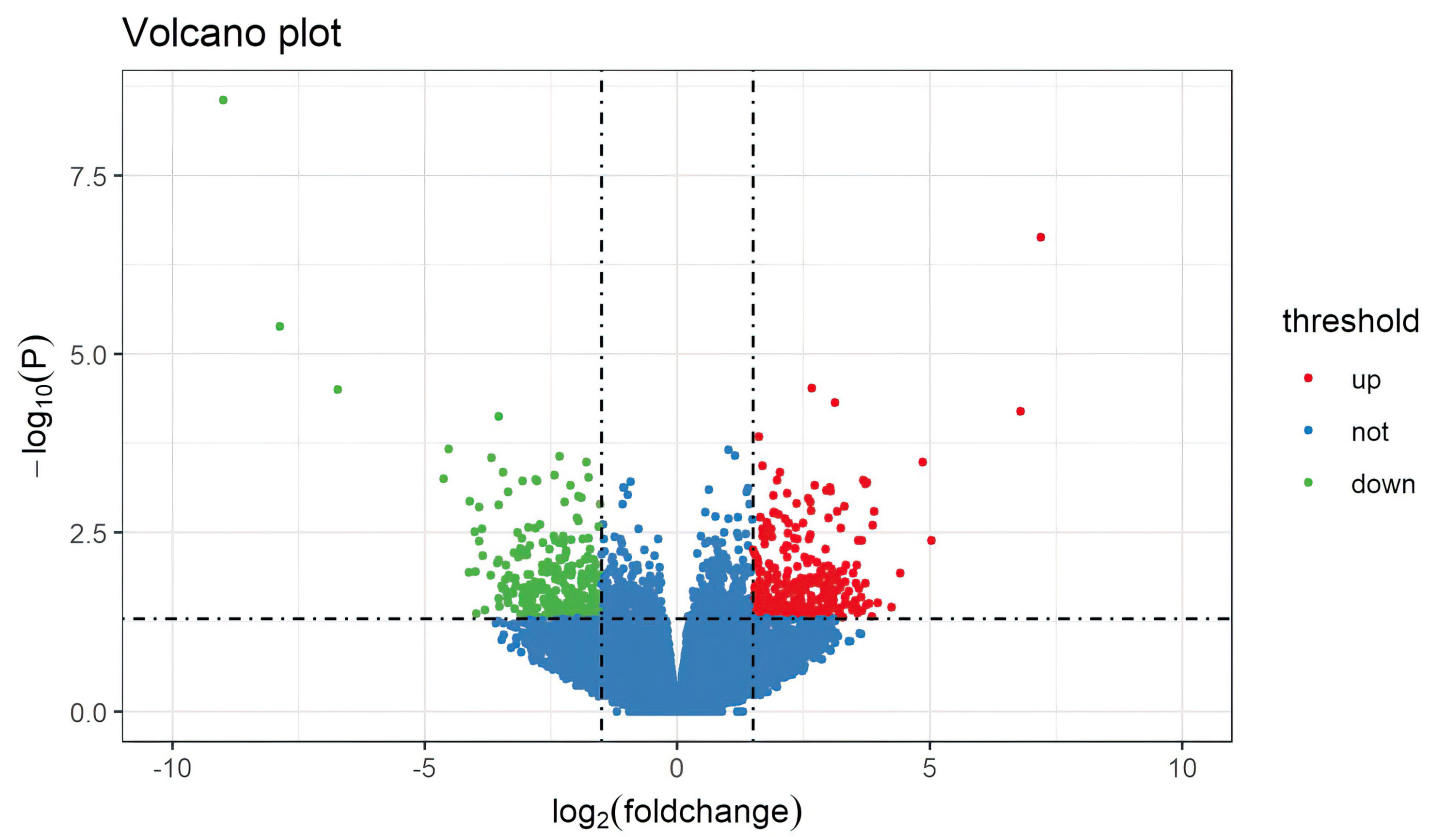
**Heatmap of differentially expressed genes**.

An expression matrix of 57,773 genes was obtained from 20 samples. Using a threshold of a *p*-value adjusted by the Benjamini-Hochberg method to less than 0.05, a total of 1019 DEGs were identified (Fig. 1). Among these DEGs, 535 were found to be up-regulated, while 484 were down-regulated. The top 5 and bottom 5 DEGs based on “log_2_FoldChange” and their respective functions are summarized in **Supplementary Table 3**. These genes include *RP11.54O7.1*, *RP4.576H24.5*, *RP4.655C5.4*, and *RP11.459O1.2*, which are long non-coding RNAs potentially involved in gene expression regulation; troponin T type 2 (*TNNT2*), which plays a critical role in myocardial contraction and is associated with cardiomyopathies; gremlin 1 (*GREM1*), which regulates bone morphogenetic protein (BMP) signaling and is involved in cell differentiation and fibrotic diseases; ribosome production factor 2 homolog (*RPF2P1*), which is essential for protein synthesis; aminopeptidase Q (*AQPEP*), which encodes a metalloproteinase involved in blood pressure regulation; actin beta pseudogene 8 (*ACTBP8*), a pseudogene of actin with potential roles in gene regulation; and glutathione S-transferase M1 (*GSTM1*), which is associated with metabolic syndrome risk, cellular detoxification, and cancer susceptibility. The more information of the DEGs is provided in **Supplementary Table 4**.

### 3.3 WGCNA Network Analysis for Gene Module and Hub Gene Identification

**Fig. 2.**
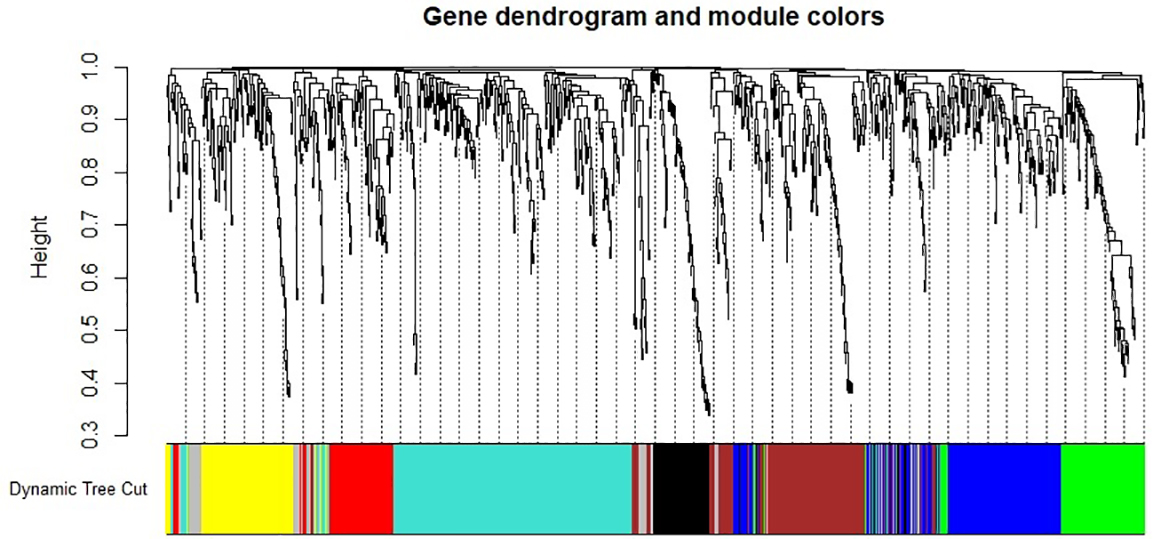
**Gene clustering tree**. The colored bars beneath the dendrogram represent module membership identified by the dynamic tree cut method. The height indicates the co-expression distance, with each vertical line representing a gene and each color corresponding to a module.

WGCNA network analysis identified a total of 8 mRNA gene modules, with sizes ranging from 55 to 271 genes. Each module is represented by a different color, and genes that could not be assigned to any module are depicted in gray (Fig. 2).

**Fig. 3.**
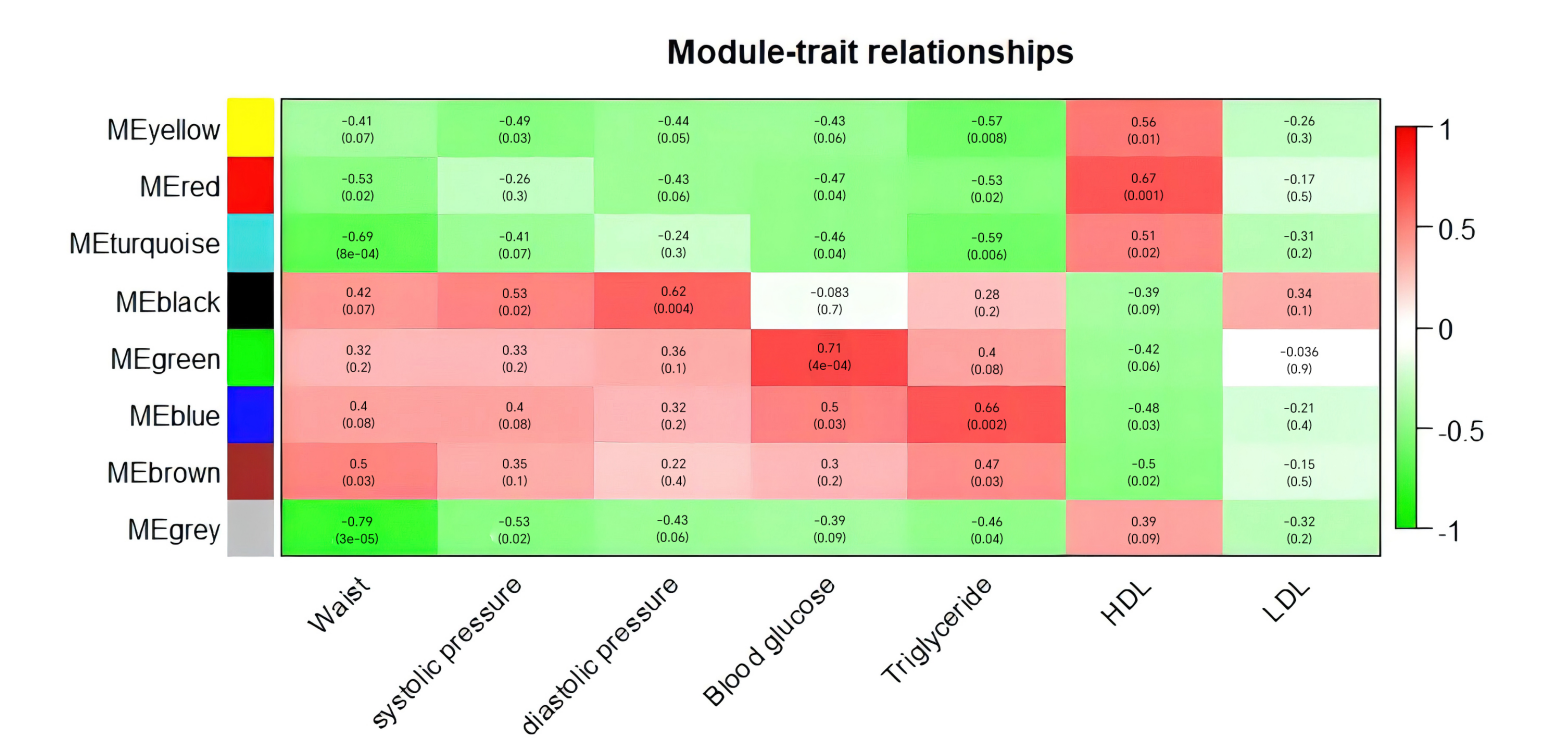
**Module-trait relationships**. Each row corresponds to a module eigengene, and each column corresponds to a phenotype of metabolic syndrome. Each cell contains the corresponding correlation coefficient and *p*-value. The table is color-coded based on correlation, as indicated by the color scale. LDL, low-density lipoprotein; ME, Module eigengene.

In the module-trait association analysis, the yellow, red, and turquoise modules were negatively correlated with metabolic syndrome, while the black, green, blue, and brown modules were positively correlated with it. Regarding the components of metabolic syndrome, the brown module was positively correlated with waist circumference and negatively correlated with HDL cholesterol. The turquoise module was negatively correlated with waist circumference and triglycerides. The black module was positively correlated with blood pressure. The green module was positively correlated with blood glucose and triglycerides. The yellow module was negatively correlated with blood pressure. The red module was negatively correlated with blood glucose and positively correlated with HDL cholesterol. No module showed a significant correlation with low-density lipoprotein (LDL) cholesterol (Fig. 3).

Finally, we selected the genes with the highest kWithin values from the 7 modules and identified them as hub genes, which wereacid phosphatase-like 2 (*ACPL2*), T cell receptor alpha variable 12-2 (*TRAV12-2*), matrix metallopeptidase 8 (*MMP8*), piggyBac transposable element derived 4 pseudogene 1 (*PGBD4P1*), transmembrane protein 261 (*TMEM261*), and brain-derived neurotrophic factor (*BDNF*) (Table 2).

**Table 2.** **Hub gene of each module**.

Module	Gene
Black	*RP11.611O2.6*
Blue	*ACPL2*
Brown	*TRAV12-2*
Green	*MMP8*
Red	*PGBD4P1*
Turquoise	*TMEM261*
Yellow	*BDNF*

*ACPL2*, acid phosphatase-like 2; *TRAV12-2*, T cell receptor alpha variable 12-2; *MMP8*, matrix metallopeptidase 8; *PGBD4P1*, piggyBac transposable element derived 4 pseudogene 1; *TMEM261*, transmembrane protein 261; *BDNF*, brain-derived neurotrophic factor.

### 3.4 PPI Network Analysis and Functional Annotation

**Fig. 4.**
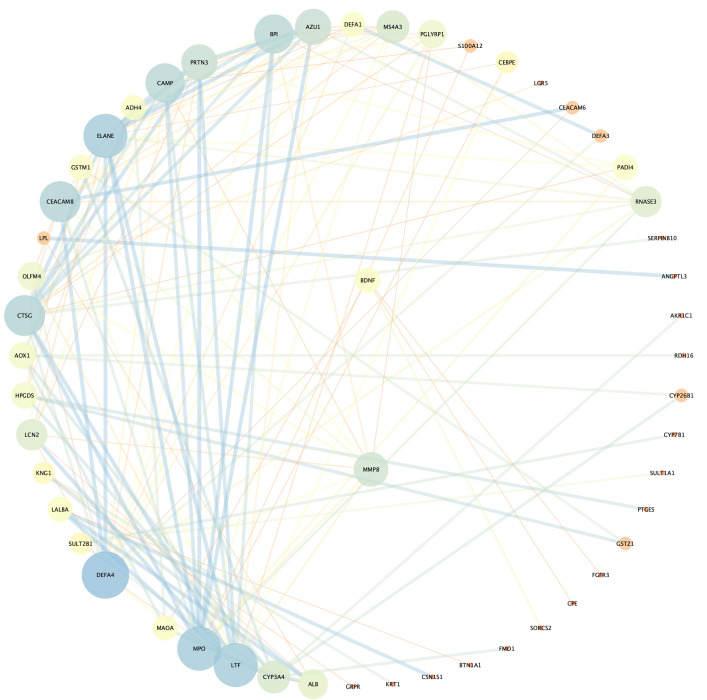
**Protein-protein interaction (PPI) network analysis**.

**Fig. 5.**
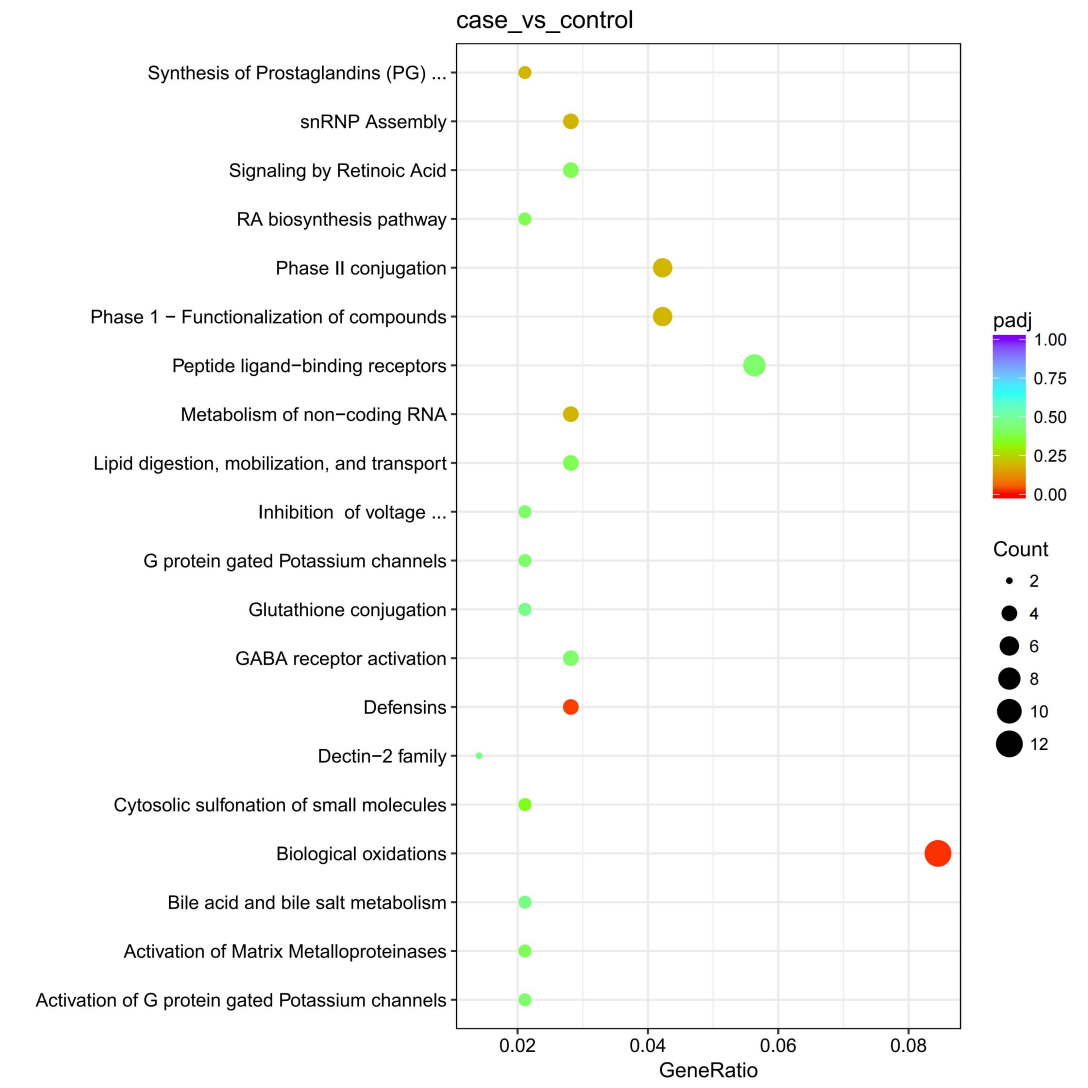
**Pathway enrichment analysis**. snRNP, small nuclear ribonucleoprotein; GABA, gamma-aminobutyric acid.

There are 386 nodes and 146 edges in PPI network (**Supplementary Table 5**). By integrating the results of PPI network analysis and WGCNA, the overlapping proteins identified were BDNF and MMP8 (Fig. 4). GO enrichment analysis and pathway enrichment analysis were conducted on the 1019 DEGs. The Reactome pathways most strongly associated with these genes included biological oxidation reactions and defensins (Fig. 5). These genes were predominantly linked to phenylalanine blue particles, primary lysosomes, secretory granules, and vesicle lumens surrounded by the cytoplasmic membrane. The biological processes associated with these DEGs encompassed immune response, morphological changes, and metabolic processes. Regarding molecular functions, the genes were involved in sulfide binding, peptidase inhibitor activity, endopeptidase regulation, voltage-gated cation channel activity, oxidoreductase activity, and glucose transmembrane transport activity. Detailed information is provided in **Supplementary Tables 6,7**. KEGG pathway analysis revealed that the DEGs were mainly associated with drug metabolism, tyrosine metabolism, steroid hormone biosynthesis, the peroxisome proliferator-activated receptor (PPAR) signaling pathway, systemic lupus erythematosus, and neuroreceptor-ligand interactions (**Supplementary Table 8**).

### 3.5 Mendelian Randomization

**Fig. 6.**
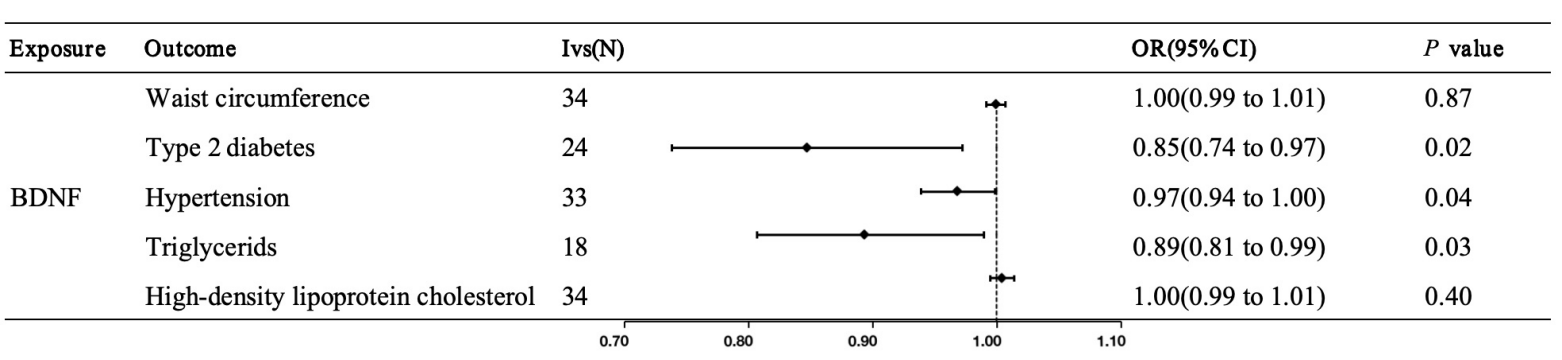
**Genetic predicted serum levels of protein BDNF on the risk of metabolic syndrome components in the MR analysis**. BDNF, brain-derived neurotrophic factor; MR, Mendelian randomization; IVs, instrumental variables; OR, odds ratio; 95% CI, 95% confidence interval.

MR analysis indicated that genetically predicted higher serum BDNF levels were causally associated with reduced risks of type 2 diabetes (odds ratio (OR) = 0.85, 95% confidence interval (95% CI): 0.74–0.97, *p* value = 0.02), hyperlipidemia (OR = 0.89, 95% CI: 0.81–0.99, *p* value = 0.03) and hypertension (OR = 0.97, 95% CI: 0.94–1.00, *p* value = 0.04; Fig. 6). There was no significant causal relationship observed between serum BDNF levels and HDL cholesterol and waist circumference (*p* value_all_ > 0.05; Fig. 6). No horizontal pleiotropy was detected in the MR-Egger analysis, confirming no violation of MR assumptions, while significant heterogeneity was observed in the Cochran’s Q test (**Supplementary Table 9**). The leave-one-out analysis exhibited no substantial influence when each SNP was individually removed (**Supplementary Fig. 2**).

## 4. Discussion

This study employed a 1:1 matched case-control design, with no statistically significant differences between the case and control groups in terms of age, gender, age of onset, total illness duration, medication duration, marital status, smoking, alcohol consumption, family history of mental illness, clozapine dosage, clozapine plasma concentration and CGI-S total score. While intergroup PANSS differences were observed, it should be emphasized that all patients were clinically stable with comparable CGI-S scores, suggesting these variations likely reflect methodological considerations rather than clinical significance. First, PANSS’s multidimensional assessment may amplify subclinical fluctuations. Second, the limited sample size (n = 20) potentially reduced statistical power, particularly given PANSS’s sensitivity to nuanced symptom dimensions. Finally, baseline heterogeneity in chronic populations could accentuate scale-specific sensitivity differences. This approach helped eliminate several confounding factors contributing to the high prevalence of metabolic syndrome in schizophrenia, making the molecular mechanism study of clozapine-induced MetS more targeted.

A total of 1019 DEGs were identified in this study. GO functional annotation and pathway enrichment analyses revealed that these DEGs are involved in several key biological processes, including immune responses (e.g., defense response to fungi [GO:0050832], defense response to bacteria [GO:0042742], and humoral immune response [GO:0006959]), cell killing activities (e.g., killing of cells of other organisms [GO:0031640], cell killing [GO:0001906]), and responses to xenobiotics (e.g., response to xenobiotic stimulus [GO:0009410], xenobiotic metabolic process [GO:0006805]). Additionally, the DEGs are implicated in processes related to symbiotic interactions, such as the defense response to other organisms [GO:0098542] and the growth of symbionts involved in interactions with the host [GO:0044116]. The KEGG pathway analysis identified significant enrichment in pathways associated with biological oxidation reactions, while the Reactome pathway analysis highlighted defensins as strongly associated with these DEGs. These findings align with prior evidence linking chronic inflammation to the pathogenesis of both schizophrenia and metabolic syndrome [21, 22]. Activation of inflammatory pathways can disrupt normal metabolic processes, thereby promoting the development of metabolic syndrome. Clozapine may induce inflammation by promoting the production of pro-inflammatory cytokines, thereby disrupting immune balance in the body. Additionally, clozapine alters mitochondrial function and increases the generation of reactive oxygen species (ROS), leading to oxidative stress and cellular damage [12, 23, 24, 25]. These mechanisms collectively may play a key role in the pathogenesis of clozapine-induced MetS.

Using WGCNA, this study identified eight gene modules and seven hub genes associated with clozapine-induced MetS, including *RP11.611O2.6*, *ACPL2*, *TRAV12-2*, *MMP8*, *PGBD4P1*, *TMEM261*, and *BDNF*. Among the identified hub genes, BDNF has been strongly linked to the pathogenesis of clozapine-induced MetS, with several studies demonstrating its role in regulating glucose metabolism, insulin resistance, and adiposity in patients treated with clozapine [26, 27]. Additionally, TMEM261 appears to have partial protective roles in the context of MetS. Studies have suggested that genes within the TMEM family can modulate insulin signaling and lipid metabolism, which may contribute to their protective effects in metabolic regulation [28, 29, 30]. Furthermore, MMP8, a gene involved in inflammation and tissue remodeling, has been identified as a risk factor for MetS, with its role in chronic inflammation and insulin resistance linking it to the development of metabolic syndrome [31]. These findings underscore the robustness of the WGCNA methodology in identifying key genes involved in the pathogenesis of clozapine-induced MetS.

BDNF, a member of the neurotrophic factor superfamily, is widely distributed throughout the central and peripheral nervous systems, with the highest concentrations found in the hippocampus and cortex. BDNF plays a crucial role in the survival, differentiation, and apoptosis of neurons, as well as in synaptic plasticity, neurotransmission, neuronal repair, and neuroplasticity in both the central and peripheral nervous systems [32]. Recent studies have also highlighted its involvement in the regulation of glucose and energy metabolism, suggesting that BDNF may contribute to metabolic disorders, including those induced by antipsychotic medications like clozapine. BDNF is involved in regulating blood glucose and energy metabolism, exhibiting a clear hypoglycemic effect. The primary receptors for BDNF are the p75 neurotrophin receptor and the tyrosine kinase receptor B (TrkB). Binding of BDNF to TrkB activates intracellular signaling cascades, enhancing TrkB phosphorylation and participating in the regulation of blood glucose and energy metabolism [27, 33].

In this study, we observed that the expression of BDNF was significantly lower in schizophrenia patients with clozapine-induced MetS compared to those without metabolic syndrome. These findings align with previous research suggesting that reduced levels of BDNF may exacerbate metabolic dysregulation [34, 35]. Animal studies have shown that *BDNF* gene knockout mice exhibit significant weight gain [36], while exogenous BDNF administration in rats leads to decreased appetite and weight loss [37]. BDNF also lowers elevated blood glucose and improves lipid metabolism, effectively preventing the progression of prediabetic mice to clinical diabetes [38, 39, 40]. Clinical studies have found reduced serum and plasma BDNF levels in type 2 diabetes patients, with changes in BDNF levels negatively correlated with insulin resistance [41, 42]. Genetic studies have primarily focused on the *BDNF *Val66Met polymorphism, where the SNP at the rs6265 locus replaces the codon for valine (Val) at position 66 with methionine (Met), impairing protein packaging and secretion of function-dependent BDNF, as well as disrupting dendritic targeting of *BDNF* mRNA [43]. Numerous clinical trials have confirmed that the *BDNF *Val66Met genotype is closely associated with weight gain, obesity, and insulin resistance [26, 44]. Overall, clozapine may influence the levels of BDNF in the brain or serum of schizophrenia patients, triggering the onset and development of metabolic syndrome.

TMEM261, also known as distal membrane arm assembly component 1 (DMAC1), is a mitochondrial inner membrane protein involved in the assembly of complex I in the mitochondrial respiratory chain. Knockout of *DMAC1* causes complex I assembly defects, which are linked to mitochondrial diseases. These defects impair core reactions in nicotinamide adenine dinucleotide hydride (NADH) dehydrogenase, electron transport, and proton pump modules, disrupting enzyme activity, altering nicotinamide adenine dinucleotide (NAD^+^)/NADH ratios, increasing reactive oxygen species (ROS) levels, and reducing adenosine triphosphate (ATP) production [30]. Elevated mitochondrial ROS levels lead to oxidative damage and an imbalance between oxidation and antioxidation in the body, favoring oxidative conditions that induce neutrophil infiltration, increased protease secretion, and the generation of oxidative intermediates, which is referred to as oxidative stress. Oxidative stress has long been implicated in the mechanisms underlying antipsychotic drug-induced metabolic syndrome or increased diabetes risk [28, 29].

MMP8 is a collagenase stored in neutrophils, released during chemotactic factor stimulation and inflammation. Studies have shown that MMP8 is positively correlated with the components of metabolic syndrome, with higher MMP8 levels associated with greater values in MetS components. MMP8 may influence the development of coronary artery syndrome by participating in inflammation [31]. *TRAV12-2 *encodes the variable region of the T-cell receptor alpha chain, which is responsible for recognizing major histocompatibility complex (MHC) and participates in cellular immunity. Numerous studies have confirmed the involvement of immune-inflammatory responses in the development of both metabolic syndrome and schizophrenia [45, 46, 47]. Therefore, antipsychotic drugs may enhance the sensitivity of the immune-inflammatory response, leading to immune dysregulation, which could be one of the mechanisms underlying clozapine-induced MetS.

Based on the above, we believe that these genes are associated with antipsychotic drug-induced metabolic syndrome, further supporting the reliability of the hub genes identified by WGCNA. While no conclusive evidence has been found linking *RP11.611O2.6*, *ACPL2*, and *PGBD4P1* to clozapine-induced MetS, the current limitations of research do not rule out the possibility of their involvement in antipsychotic drug-induced metabolic syndrome.

The innovation of this study lies in the 1:1 matched case-control design, which eliminates many confounding factors contributing to the high prevalence of metabolic syndrome in schizophrenia, such as genetic background, age, gender, illness duration, medication regimen, medication duration, and lifestyle factors, making the molecular mechanism study of clozapine-induced MetS more focused. Secondly, by using gene chip data, which differs from previous single-gene analyses, we can obtain global information on all gene transcripts under specific conditions, helping to uncover the molecular mechanisms and transcriptional regulatory patterns of clozapine-induced MetS. Lastly, WGCNA was used to conduct in-depth analysis and mining of the transcriptomic data, providing clinical researchers with valuable scientific evidence for preventing and treating metabolic syndrome during the treatment of mental illnesses.

However, there are several limitations in this study. First, we used peripheral blood leukocytes to study gene mRNA expression levels, which cannot fully replace gene expression in brain tissue. Second, not all participants were exclusively taking clozapine. Although we excluded patients on adjunctive antipsychotics (e.g., olanzapine, risperidone, quetiapine, or amisulpride) in sensitivity analyses and confirmed no group differences in clozapine dosage (*p* = 0.896) or plasma levels (*p* = 0.325; Table 1), residual confounding from prior polypharmacy cannot be fully excluded. Specifically, these adjunctive agents may independently induce metabolic dysregulation [3, 13] and indirectly amplify clozapine’s toxicity through cytochrome P450 family 1 subfamily A polypeptide 2 (CYP1A2)/cytochrome P450 family 3 subfamily A polypeptide 4 (CYP3A4) inhibition, thereby altering pharmacokinetics [48]. Notably, RNA sequencing analysis of clozapine-monotherapy patients identified DEGs that were significantly enriched in immune response pathways (**Supplementary Table 10, Supplementary Fig. 3**), consistent with the full cohort findings. Third, both schizophrenia and metabolic syndrome are the result of complex gene-environment interactions, and this study only analyzed 10 pairs of samples in a cross-sectional manner, lacking pre- and post-medication transcriptomic comparisons and with a small sample size. Future studies should aim to adopt stricter inclusion criteria, expand sample size, conduct long-term follow-ups, and integrate genomic and proteomic technologies to develop more rigorous experimental designs for large-sample repeated experiments.

## 5. Conclusion

This study identified seven hub genes associated with clozapine-induced MetS in schizophrenia patients through WGCNA and transcriptomic analysis, including *RP11.611O2.6*, *ACPL2*, *TRAV12-2*, *MMP8*, *PGBD4P1*, *TMEM261*, and *BDNF*. Among these, *RP11.611O2.6*, *MMP8*, *TRAV12-2*, and *ACPL2* were upregulated, whereas *PGBD4P1*, *TMEM261*, and *BDNF* were downregulated. Functional annotation revealed that these genes are potentially involved in key pathways related to inflammation, mitochondrial dysfunction, and metabolic regulation, providing new insights into the molecular mechanisms underlying clozapine-induced MetS.

BDNF is suggested as a potential biomarker due to its involvement in glucose metabolism, energy balance, and neurotrophic functions, as supported by prior studies. However, we acknowledge that further functional validation is required to confirm its role in clozapine-induced MetS. Similarly, MMP8, TMEM261 and TRAV12-2 have been linked to metabolic and inflammatory pathways, further research is needed to validate their specific roles in this context. The application of WGCNA in identifying relevant gene modules highlights its value in investigating gene-environment interactions in psychiatric and metabolic disorders.

Despite limitations such as a small sample size and the use of peripheral blood mRNA as a proxy for tissue-specific expression, the study offers new perspectives on the pathophysiology of clozapine-induced MetS and its clinical management. Future research should explore these mechanisms further using multi-omics approaches and larger sample sizes.

## Availability of Data and Materials

The data that support the findings of this study are available from the corresponding author upon reasonable request.

## Supplementary Material

Supplementary material associated with this article can be found, in the online version, at https://doi.org/10.31083/AP49352.
