# Supplementary material for: Integrative Bioinformatics Analysis Reveals Pathogenesis Biomarkers for Clozapine-Induced Metabolic Syndrome
Source: Alpha Psychiatry. 2025 Dec 22;26(6):49352. doi: 10.31083/AP49352 (PMC12781211; doi:10.31083/AP49352)
Supplement: Supplementary file 1 [file 2757-8038-26-6-49352-s1.zip › Supplementary Material.docx]

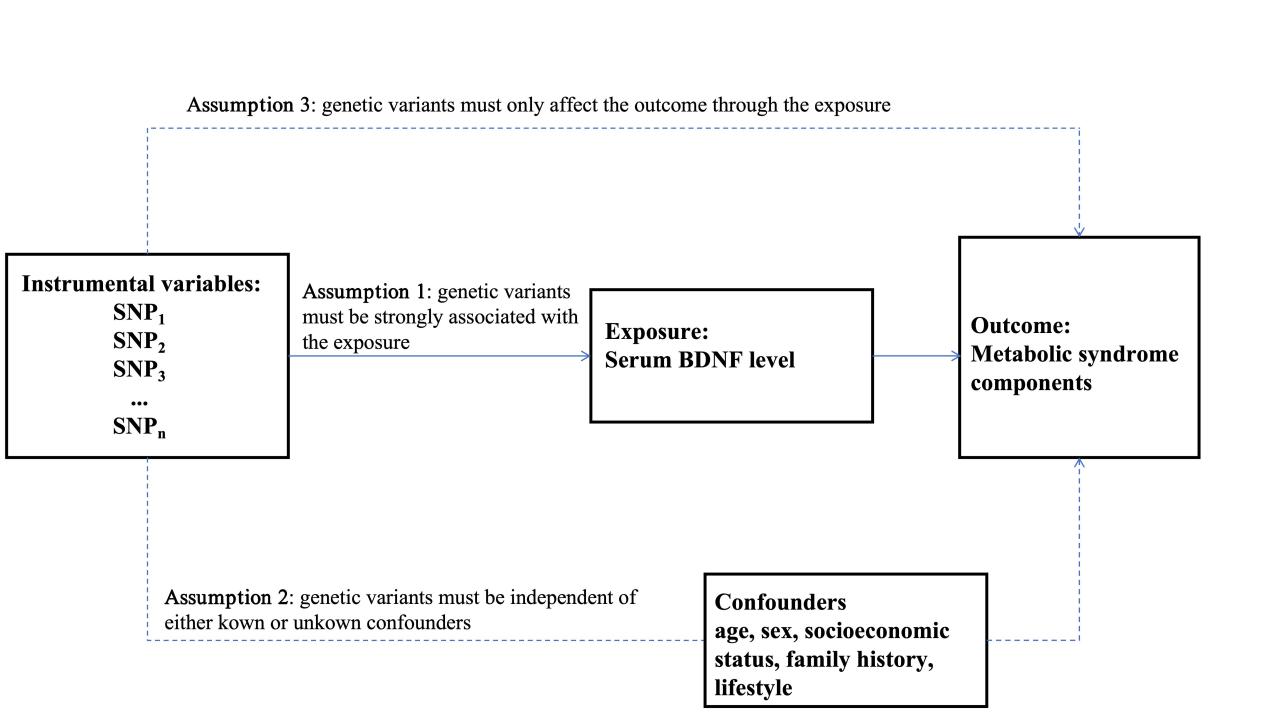


**Supplementary Fig. 1. Schematic diagram of the MR assumptions.**


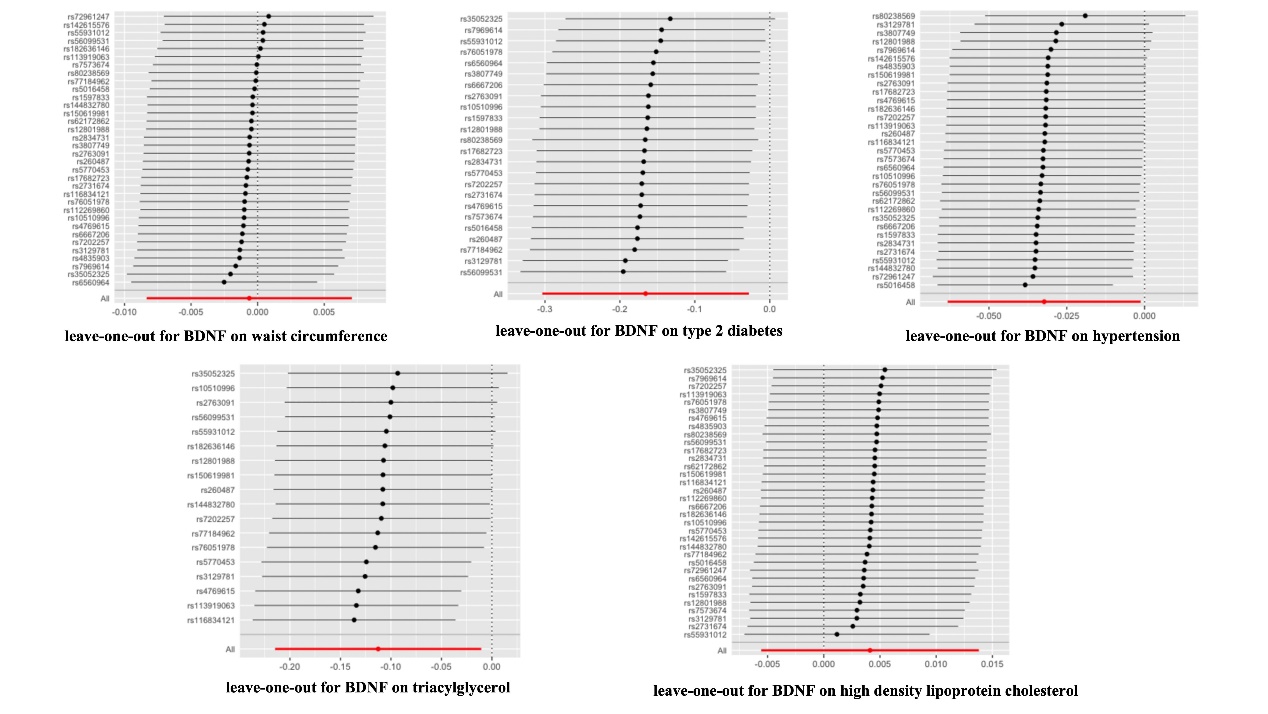


**Supplementary Fig. 2. Leave-one-out analysis of the association between serum BDNF level and metabolic syndrome components genetically in the MR analysis.**


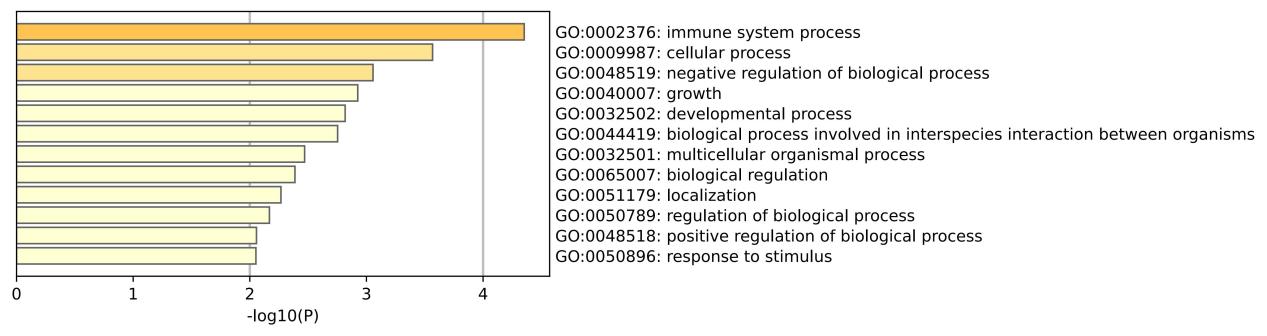


**Supplementary Fig. 3. Enriched GO Biological Processes in Clozapine-Monotherapy MetS vs. Non-MetS.**
